# Tobacco industry’s use of outer packaging in Mexico increases product marketing space

**DOI:** 10.18332/tpc/179633

**Published:** 2024-02-01

**Authors:** Reiley Hartmuller, Graziele Grilo, Joanna E. Cohen, Kevin Welding, Luz Myriam Reynales-Shigematsu, Katherine Clegg Smith

**Affiliations:** 1 Institute for Global Tobacco Control, Department of Health, Behavior and Society, Johns Hopkins Bloomberg School of Public Health, Baltimore, United States; 2 Departamento de Prevención y Control de Tabaquismo, Centro de Investigación en Salud Poblacional, Instituto Nacional de Salud Pública, Cuernavaca, Morelos, Mexico; 3 Department of Health, Behavior and Society, Johns Hopkins Bloomberg School of Public Health, Baltimore, United States

**Keywords:** tobacco packaging, outer packaging, cigarettes, packaging policy, tobacco advertising, low- and middle-income countries

**Dear Editor,**

Mexico requires graphic health warning labels (HWLs) covering 30% of the front, and text HWLs on 100% of the back and one side of cigarette packs^1^. The tobacco industry has used inserts and onserts (information attached to packaging) to increase marketing space on packs^2^. Companies can also include marketing on outer packaging that encloses the primary package that directly contains the cigarettes^3^. Outer packaging provides additional space for advertising and promotion and can obscure HWLs^4^. For example, Japan Tobacco International introduced a limited-edition tin pack before the implementation of plain packaging in Ireland, seemingly to reduce policy impact^5^. Here, we describe marketing appeals on Mexican cigarette outer packaging that potentially undermine packaging policy.

Following the Tobacco Pack Surveillance System (TPackSS) systematic protocol^6^, unique cigarette packs (i.e. packs with at least one different exterior feature, such as color or stick count) were purchased in Mexico during October–November 2021. Purchases were made in twelve culturally and geographically diverse neighborhoods, of different socioeconomic status, in five cities (Mexico City, Guadalajara, León, Durango, and Mérida). Packs were purchased from convenience stores, small/independent grocery stores, wholesalers, and pharmacies, then photographed and double-coded, by three independent coders, for the presence of various marketing appeals.

Two hundred sixty-two unique cigarette packs were purchased. Of these, 24 contained outer packaging, and 12 (50%) outer packs were of a box/tin style, as opposed to sliders; 10 (42%) of the outer packs presented limited-edition descriptors. Seven (29%) presented Mexican national appeal imagery or descriptors, such as the image of a ‘lucha libre’ (wrestling) mask, and 4 (17%) indicated they could be used as an ashtray.

A common theme for outer packaging was to announce a packaging change; 10 (42%) outer packs advertised a change to the appearance of the primary pack (Figure 1).

**Figure 1:**
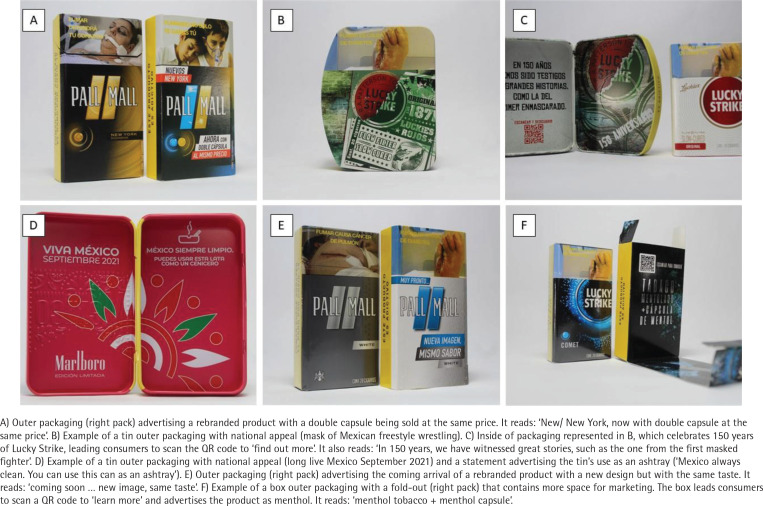
Examples of cigarette outer packaging, Mexico, 2021

Product information on the outer packaging differed from the primary pack in several cases. One Pall Mall outer pack conveyed higher levels of tar, nicotine, and carbon monoxide compared to what was stated on the primary pack. Two Marlboro outer packs had zeroes for tar, nicotine, and carbon monoxide – misleading information not on the primary pack. A Winston outer pack advertised a different flavor than what was presented on the primary pack. Most outer packs (63%) also directed people online, such as the company website, and/or QR codes.

In Mexico, outer packaging demonstrates a range of marketing appeals and could be a way for tobacco companies to counteract current HWL regulations that help limit marketing and advertising space on packaging. They may also mislead consumers on content emissions. Packs designed as metal tins also present the opportunity to be kept as collectors’ items, and advertising their use as ashtrays encourages consumers to keep them, extending exposure to cigarette promotional messaging. These packs are the same price as without the tins, such that the tin is a free gift with each purchase. These findings stress the importance of plain and standardized packaging, including eliminating outer packaging, to protect consumers from appealing marketing and misleading claims.

## Data Availability

Data sharing is not applicable to this article as no new data were created.
